# MHC-II^+^ G-MDSC expressing CD80, CD86, or ICOSL are associated with pro-inflammatory CD4^+^ T cells phenotypes in the lungs of lupus-prone mice

**DOI:** 10.3389/fimmu.2026.1891967

**Published:** 2026-10-26

**Authors:** Felipe R. Uribe, Sebastián A. Echeverría-Araya, Grabiel J. García-Velázquez, Karissa Chávez-Villacreses, Benjamín Díaz-López, Valeria Salazar-Montoya, Alison Sepúlveda-Pontigo, Jocelyn Tobar, Felipe Simon, Rodolfo Paredes, Felipe Melo-González, Katina Schinnerling, Juan P. Mackern-Oberti, Abel E. Vasquez, Karen Bohmwald, Jorge A. Soto

**Affiliations:** 1 Laboratory of Virology and Integrative Translational Immunology, Faculty of Life Sciences, Universidad Andres Bello, Santiago, Chile; 2 Center for Research on Pandemic Resilience, Faculty of Life Sciences and Institute of Public Health, Universidad Andres Bello, Santiago, Chile; 3 Millenium Institute of Immunology and Immunotherapy, Santiago, Chile; 4 Veterinary School, Faculty of Life Science, Universidad Andres Bello, Santiago, Chile; 5 One Health Institute, Faculty of Life Sciences, Universidad Andres Bello, Santiago, Chile; 6 Translational Immunology Laboratory, Faculty of Life Sciences, Universidad Andres Bello, Santiago, Chile; 7 Universidad Nacional de Cuyo, Faculty of Medical Sciences, Mendoza, Argentina; 8 Instituto de Medicina y Biología Experimental de Cuyo (IMBECU), Centro Científico Tecnológico (CCT), Consejo Nacional de Investigaciones Científicas y Técnicas (CONICET), Mendoza, Argentina; 9 School of Veterinary Medicine, Faculty of Medicine and Health Sciences, Universidad Mayor, Santiago, Chile; 10 Center for Biomedicine, Universidad Mayor, Santiago, Chile; 11 Instituto de Ciencias Biomédicas, Facultad de Ciencias de la Salud, Universidad Autónoma de Chile, Santiago, Chile

**Keywords:** CD4+ T cells, cellular infiltration, G-MDSCs, lung, MHC-II, M-MDSCs, SLE

## Abstract

Systemic lupus erythematosus (SLE) is a chronic inflammatory autoimmune disease that affects various tissues of the body. One of these tissues is the lung, in which pleuropulmonary manifestations commonly occur. In these patients, homeostasis is disrupted, characterized by a decrease in T_reg_ and an increase in T_h1_ and T_h2_ cells. Myeloid-derived suppressor cells (MDSCs) are a cell population characterized by their ability to suppress the T cell response; however, in recent years, the presence of major histocompatibility complex class II (MHC-II) molecules on MDSCs has been reported. Nevertheless, the presence of costimulatory molecules, such as CD80, CD86, and ICOSL, has not been studied yet in MDSCs expressing MHC-II in the context of SLE. In this study, we evaluated the pulmonary microenvironment in SLE mice from an immunological and histological perspective. We observed increased pro-inflammatory cytokines and cellular infiltration in lungs of SLE mice. We also observed MDSC MHC-II cells expressing CD80, CD86, and ICOSL in this tissue, along with alterations of CD4^+^ T cells subsets, characterized by a decrease in IL-10 frequency in SLE mice. Moreover, we observed that G-MDSCs MHC-II expressing CD80, CD86, or ICOSL correlate positively with phenotypes of CD4^+^ T cells that produce IL-17 and IFN-γ. For this reason, we suggest that MHC-II^+^ G-MDSC cells may contribute to an inflammatory environment that triggers tissue damage in the lungs of SLE mice.

## 1 Introduction

Systemic lupus erythematosus (SLE) is an inflammatory, chronic autoimmune disease that, unlike other autoimmune diseases, SLE affects several tissues (1, 2). It is estimated that this pathology affects 3.41 million people worldwide, ranging from children to older adults; being women the most affected, with a ratio of up to 13:1 compared to men (3, 4). The most common clinical manifestations in SLE patients include inflammatory arthritis, nephritis, cutaneous lupus, serositis, hematologic disorders, and alopecia (5). However, around 20-90% of SLE patients exhibit pleuropulmonary manifestations, with pleuritis the most common clinical presentation (6). while the less common manifestations include acute lupus pneumonitis, diffuse alveolar hemorrhage, and SLE-associated interstitial lung disease. It should be noted that these symptoms usually occur in adult patients (6, 7). From an immunological perspective, SLE patients with lung conditions have been reported to exhibit decreased regulatory T cells (T_reg_) and increased T_h1_ and T_h2_ populations in peripheral blood (7). Furthermore, an increase in CD3 T cells has been observed in the lungs of a SLE murine model, along with the presence of various myeloid cells, such as monocytes, dendritic cells, and neutrophils (8).

Myeloid-derived suppressor cells (MDSCs) are a type of myeloid cell whose relevance has gained attention in recent years, as these cells are implicated in several diseases, including lung pathologies and autoimmune diseases (9–11). Particularly in SLE mice models, MDSCs play a multifaceted role, since they have been observed to induce a protective immunoregulatory effect, but have also been shown to promote disease progression (9, 12). MDSCs in mice exhibit a granulocytic (G-MDSC) and monocyte (M-MDSC) phenotype (10). Both G-MDSCs and M-MDSCs exhibit immunosuppressive effects, primarily by modulating T cell activation and effector functions (9, 13). In mouse models of cancer and autoimmune diseases such as experimental autoimmune encephalomyelitis (EAE) and SLE, the presence of major histocompatibility complex class II (MHC-II) molecules on the surface of MDSCs has been reported (14–16). Specifically, in SLE, MDSCs expressing MHC-II have been reported in secondary lymphoid organs (14). However, the presence of costimulatory molecules on the surface of the previously mentioned cells was not evaluated in SLE condition (14). Co-stimulatory molecules such as CD80, CD86, and ICOSL, among others, play a crucial role in SLE development, since they are associated with antigen-presenting cell (APC) function, T-cell activation and autoantibody production, which lead to inflammation and tissue damage (17, 18).

Based on this information, in this article we evaluate the lungs histological and immune environment in an SLE mouse model context, focusing specifically on MDSCs expressing MHC-II and CD4^+^ T cells. Our results indicate the establishment of an inflammatory environment in the lungs of SLE mice, characterized by increased cellular infiltration and elevated histopathological scores consistent with lung injury. In addition, we identified MHC-II^+^ G-MDSC cells expressing CD80, CD86, or ICOSL that correlates with phenotypes of CD4^+^ T cells that produce IL-17 and IFN-γ. These findings suggest that G-MDSCs with an APC-like phenotype may contribute to the development of lung inflammation and tissue damage in SLE.

## 2 Materials and methods

### 2.1 Mice

We used male and female MRL/MpJ (control) and MRL/MpJ-Fas*^lpr^*/J (SLE) mice, obtained from The Jackson Laboratory (Bar Harbor, ME, USA). Animals were housed under specific pathogen-free conditions in the institutional animal facility at a constant temperature of 23 °C, with 12-h light/dark cycles and access to food and water *ad libitum*. Euthanasia was performed from 16 weeks old through an intraperitoneal injection of a ketamine (80 mg/kg) and xylazine (4 mg/kg) mix. All procedures were performed accordance with the animal handling manual of the Bioethics Committee of the Universidad Andrés Bello (09/2024).

### 2.2 Sample collection

To quantify anti-dsDNA antibodies, blood samples the blood samples were collected by cardiac puncture, which was deposited without anticoagulant in a thermoregulated bath at 37 °C for 30 minutes. Afterwards, the samples were centrifuged at 2200 x g for 5 minutes at 21 °C, and the serum was recovered.

Lung samples were collected from mice and divided into different sections for analysis. For flow cytometry, the lung tissue (right lung, accessory and caudal lobes) was treated with 2 mg/mL of collagenase type IV (Gibco, cat# 17104-019) and disaggregated using surgical scissors. Subsequently, the samples were incubated with shaking at 225 rpm and 37 °C for 1 hour; following incubation, PEB 1x (0.5% bovine serum albumin (Rockland, cat# 001-0133), 2 mM EDTA (Invitrogen, cat# AM9261), and PBS 1x at pH 7.4) was added to stop the digestion reaction. Afterwards, the tissues were mechanically homogenized using a 70 μm cell strainer and centrifuged at 300 x g for 5 min at 4 °C. To remove erythrocytes, 2 mL of RBC Lysis Buffer 1x (Biolegend, cat #420301) was added and incubated for 5 min at room temperature (RT); subsequently, PBS 1x was added to stop the reaction, followed by centrifugation at 300 x g for 5 min at 4 °C to obtain the tissue cells.

To obtain proteins, the tissue (right lung, cranial and middle lobes) was placed in 500 μL of RIPA Lysis Buffer 1x (Merck, cat# 20-188) supplemented with complete Mini EDTA-free 1X (Merck, cat# 11836170001) and homogenized using a D1000 Hand-held Homogenizer (Benchmark Scientific). The sample was centrifuged at 12,000 x g for 5 min, and the supernatant was collected. Finally, the non-lobulated segment of the lung was recovered for histology; these tissues were fixed in All-Fix (Cancer Diagnostics Inc, cat# FX1073) at RT.

### 2.3 Quantification of anti-dsDNA antibodies and cytokines

To measure anti-dsDNA antibodies, an in-house ELISA was performed. The procedure involved adding 0.1 mg/mL of calf thymus DNA antigen (Invitrogen, cat# 15633-019) to each well and incubating overnight at 4 °C. The plate was then blocked with 1x PBS + 10% FBS for 2 hours with shaking. For the calibration curve, a dilution was prepared starting from 500,000 pg/mL of anti-dsDNA antibody (Abcam, cat#AB27156), while for the samples a 1:500 dilution was made; both were incubated for 2 hours at room temperature with shaking. Subsequently, a 1:2,000 dilution of the secondary antibody HRP goat anti-mouse IgG (Invitrogen, cat# 62-6520) was added and incubated for 50 min. Afterward, 100 μL of TMB (BioLegend, cat# 421101) was added for 5 min, and the reaction was stopped with 50 μL of stop solution (Invitrogen, cat# SS04). Finally, the plate was read in a spectrophotometer, Multiskan FC Microplate Photometer (ThermoFisher Scientific), at a wavelength of 450/570 nm.

To quantify IL-10 (BD OptEIA, cat# 555252), IL-6 (BD OptEIA, cat# 555240), TNF (BD OptEIA, cat# 558534), and IL-17A (BioLegend MAX Deluxe, cat# 432504), we followed the manufacturer’s instructions. The samples were diluted 1:2, and the plates were measured in a spectrophotometer, Multiskan FC Microplate Photometer (ThermoFisher Scientific), at a wavelength of 450/570 nm.

### 2.4 Histological analysis

The fixed lungs were dehydrated and transferred to the TP1020 automatic tissue processor (Leica Biosystems). Subsequently, the tissues were embedded in paraffin utilizing MEDITE TES Valida (Medite Medical GmbH). The tissue was sectioned into 5-μm-thick serial sections using a Leica RM2245 semi-automatic rotary microtome (Leica Biosystems). Once the sections were assembled on the slides, they were deparaffinized with xylene, followed by rehydration through a decreasing ethanol gradient. Finally, the samples were stained with hematoxylin and eosin (H&E).

The histological images were acquired by scanning the full slide at 20x using a Leica DM3000 microscope, combined with Microvisioneer and ManualWS v.2020C-34 FL software. Histological analysis was performed by a blinded observer using QuPath v.0.6.0. We developed a histopathological score system based on previously established histopathological criteria, incorporating four independent parameters, which consists of four independent parameters: I) number of inflammatory foci, II) mean inflammatory focus area (µm²), III) inflammatory cellularity (cells/focus), and IV) alveolar collapse (19–22). Each of these parameters was assigned a score on a scale of 0 to 4 (**Supplementary Table 1**). The total score per sample was obtained by summing the individual values.

### 2.5 Co-culture

We obtained the spleen, which was mechanically homogenized using a 70 μm cell strainer and centrifuged at 300 x g for 5 min at 4 °C. To remove erythrocytes, 2 mL of RBC Lysis Buffer 1x (Biolegend, cat #420301) was added and incubated for 5 min at RT; subsequently, PBS was added to stop the reaction, followed by centrifugation at 300 x g for 5 min at 4 °C to obtain total splenocytes. Afterwards, we used columns to isolate MDSCs (Miltenyi Biotec, cat# 130-094-538) and CD4^+^ T cells (Miltenyi Biotec, cat# 130-104-454), following the manufacturer’s instructions. Once we had isolated these cells, we performed a co-culture between them. The culture conditions were as follows: I) CD4^+^ T cells in supplemented RPMI 1640 (10% FBS, Antibiotic-Antimycotic 1x (Gibco, cat#15240062), GlutaMAX 1x (Gibco, cat#35050061), 50 μM β-mercaptoethanol (Gibco, cat#31350010)), II) CD4^+^ T cells stimulated with concanavalin A (ConA) (1 μg/mL), III) CD4^+^ T cells + G-MDSCs (4:1 ratio), and IV) CD4^+^ T cells + M-MDSCs (4:1 ratio). The cells were incubated in flat-bottomed 96-well plates at 37 °C with 5% CO_2_ for 2 days.

### 2.6 Flow cytometry

Two flow cytometry panels were performed: to MDSCs and T Cells (**Tables 1**–**5**). The cells were cultured naturally without any *ex vivo* stimulation. The cells were incubated with a viability stain (BD Horizon Fixable Viability Stain 700, cat# 564997) at 4 °C in dark for 15 minutes. Subsequently, the extracellular antibody mix (1:500 dilution in PEB 1x per antibody) was added, and the cells were incubated for 45 minutes at 4 °C in darkness. The cells were then fixed and permeabilized using the BD IntraSure kit (BD Biosciences, cat# 641776), following the manufacturer’s instructions. Intracellular antibodies (1:500 dilution in PermWash per antibody) to staining transcription factors and cytokines were added and incubated overnight in the dark at 4 °C. Finally, the cells were washed and resuspended in PBS 1x. Data were acquired using the BD FACSymphony A1 Cell Analyzer (BD Biosciences). A total of 100,000 events per animal were acquired, the data obtained were subjected to a quality analysis using PeacoQC v1.5. Dimensionality reduction was performed using Uniform Manifold Approximation and Projection (UMAP) v4.1.1, we used the selected live cells as equivalent nodes and performed a concatenation of all events. The UMAP analysis was performed jointly using samples from SLE mice and control mice. FlowSOM v4.1 was used to generate the clusters, in which 8 metaclusters were generated; their random seeds are automatically generated each time the plugin is run. Finally, each cluster was identified using the cluster explorer incorporated in FlowJo v10.10.

**Table 1:** Panel MDSCs.

Antibody	Flurochrome	Clone	Source
CD11b	APC-Cy7	M1/70	BD Biosciences
Ly6C	APC	AL-21	BD Biosciences
Ly6G	BV786	1A8	Invitrogen
MHC-II	BV421	M5/114,15,2	BioLegend
CD80	FITC	16-10A1	BioLegend
CD86	PerCP-Cy5.5	GL-1	BioLegend
ICOSL	PE	HK5.3	BioLegend
Arg-1	PE-Cy7	A1exF5	Invitrogen
iNOS	PE-Cy5.5	CXNFT	Invitrogen

**Table 2:** Panel MDSCs for exclusion markers.

Antibody	Flurochrome	Clone	Source
CD11b	APC-Cy7	M1/70	BD Biosciences
Ly6C	BV605	AL-21	BD Biosciences
Ly6G	BV786	1A8	Invitrogen
CD11c	PE-Cy7	HL3	BD Biosciences
F4/80	APC	T45-2342	BD Biosciences

**Table 3:** Panel T Cells for lung cells.

Antibody	Flurochrome	Clone	Source
CD3	BV711	145-2C11	BD Biosciences
CD4	BV650	GK1.5	BD Biosciences
CD8	PE-Cy5.5	53-6.7	Invitrogen
RORyt	PerCP	600380	R&D Systems
FOXP3	AF488	150D	BioLegend
T-Bet	PE-Cy7	4B10	BioLegend
IFN-y	PE-CF594	XMG1.2	BD Biosciences
IL-17	BV786	TC11-18H10.1	BD Biosciences
IL-10	BV605	JES5-16E4	BD Biosciences

**Table 4:** Panel T Cells for co-culture.

Antibody	Flurochrome	Clone	Source
CD4	BV650	GK1.5	BD Biosciences
RORyt	PerCP	600380	R&D Systems
FOXP3	AF488	150D	BioLegend
T-Bet	PE-Cy7	4B10	BioLegend
IFN-y	PE-CF594	XMG1.2	BD Biosciences
IL-17	BV786	TC11-18H10.1	BD Biosciences
IL-10	PE	JES5-16E3	BioLegend

**Table 5:** Panel T Cells for B220 and TCRβ^+^.

Antibody	Flurochrome	Clone	Source
CD3	BV711	145-2C11	BD Biosciences
CD4	BV650	GK1.5	BD Biosciences
CD8	PE-Cy5.5	53-6.7	Invitrogen
B220	PE	RA3-6B2	BD Biosciences
TCRβ^+^	PerCP-Cy5.5	H57-597	BD Biosciences

The gating strategy used for MDSCs was based on published works (14, 23–25), meanwhile the selection of CD4^+^ T cells is described in **Supplementary Figure 1**.

### 2.6 Statistical analysis

Statistical analysis was performed using Prism 11 software (GraphPad Software, Inc., San Diego, CA, USA). We used Welch’s t-test, one-way ANOVA post Tukey´s test, and two-way ANOVA post Tukey´s test; whose values are presented as mean ± standard error of the mean (SEM). On the other hand, we used the Mann-Whitney test for nonparametric analyses related to the histopathological score. Furthermore, for the correlation analysis, we used Pearson’s correlation coefficient with the R packages “ggplot2” v2.0.3 and “Hmisc” v5.2 in R Studio. Values with a *p-value* < 0.05 were considered statistically significant.

## 3 Results

### 3.1 The lungs of SLE mice show elevated concentrations of pro-inflammatory cytokines coupled with increased histopathological score

The MRL/MpJ-Fas^lpr^/J (SLE) animals utilized in this study exhibited clinical manifestations resembling those observed in SLE (**Supplementary Figure 2**). To evaluate potential dysregulation of the pulmonary microenvironment in these mice, we determined the concentration of different pro- and anti-inflammatory cytokines in lung tissue. We observed that in SLE animals, the lungs exhibited increased concentrations of pro-inflammatory IL-6, IL-17, and TNF (**Figures 1A, C, D**, respectively), while the concentration of IL-10 is decreased compared to control animals (**Figure 1B**).

**Figure 1:**
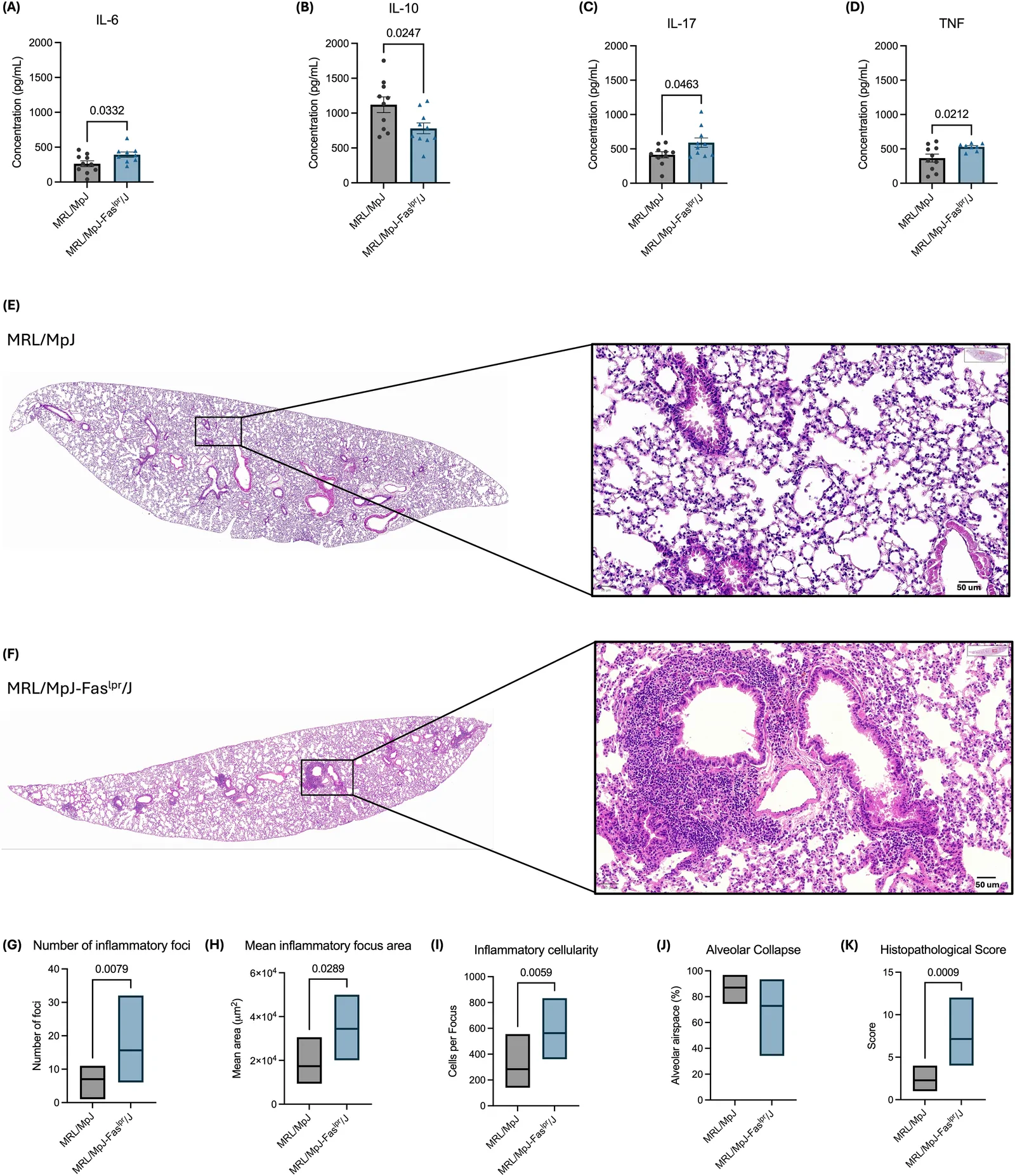
The pulmonary environment in SLE mice is exacerbated by an inflammatory response and cellular infiltration. Concentrations of **(A)** IL-6, **(B)** IL-10, **(C)** IL-17, and **(D)** TNF in the lungs of both experimental groups through ELISA. MRL/MpJ (black circles) n=10, MRL/MpJ-Fas^lpr^/J (blue triangles) n=8–10. The statistical analysis used was Welch’s t-test, p< 0.05 are considered significant. 20X scan of histological lung sections stained with Hematoxylin & Eosin, along with a zoom of areas near the bronchi of **(E)** MRL/MpJ and **(F)** MRL/MpJ-Fas^lpr^/J animals. Parameters for **(G)** Number of inflammatory foci, **(H)** Mean inflammatory focus area, **(I)** Inflammatory cellularity, **(J)** Alveolar collapse, and **(K)** Histopathological Score in each of the experimental groups. The scale bar is black and can be seen in the lower-right corner; its dimension is 50 µm. MRL/MpJ (black box plots) n=7, MRL/MpJ-Fas^lpr^/J (blue box plots) n=8. The statistical analysis used was the Mann-Whitney test, *p<* 0.05 are considered significant.

Furthermore, we evaluated whether the lung tissue exhibited histopathological alterations, either due to disruption of pulmonary architecture or the presence of cellular infiltration. H&E staining was performed on the lungs of both experimental groups (**Figures 1E, F**). We observed in the lungs of SLE mice an increase in the number of inflammatory foci, mean inflammatory focus area (µm²), and inflammatory cellularity (**Figures 1G–I**, respectively). However, the alveolar collapse was similar between both experimental groups (**Figure 1J**). Based on each of these individual parameters into the composite histopathological scoring system revealed a significantly higher lung injury score in SLE mice (**Figure 1K**). These results suggest that a disturbance of pulmonary environment in SLE animals, characterized by an increase in pro-inflammatory cytokines and increased cellular infiltration, which may promote a constant loop that contributes to lung damage and dysfunction.

### 3.2 The lungs of SLE mice present a higher frequency of M-MDSCs expressing MHC-II

The cellular infiltration observed in pulmonary tissue of SLE mice prompted us to investigate whether MDSCs expressing MHC-II, previously described in secondary lymphoid organs (spleen and axillary lymph nodes), can be found in a target organ such as the lungs of SLE mice. Nevertheless, since these cells are known to be present in secondary lymphoid organs, we first evaluated MDSCs in the spleen along with their immunosuppressive capacity over T cells. Firstly, we observed the presence of both G-MDSCs and M-MDSCs in the spleens of SLE mice (**Supplementary Figure 3A**), whose frequency was similar in both experimental groups (**Supplementary Figure 3B**). Both phenotypes express immunosuppressive molecules (Arg-1 and iNOS) characteristic of these populations (**Supplementary Figures 3C–F**); however, M-MDSCs from the SLE group show an increased frequency of iNOS (**Supplementary Figure 3F**). Regarding immunosuppressive activity, we observed that both G-MDSCs and M-MDSCs reduce the frequency of CD4^+^FOXP3^+^ T cells (**Figure 2A**). However, the presence of MDSCs does not affect the frequency of CD4^+^RORγt^+^ T cells and CD4^+^T-bet^+^ T cells (**Figures 2B, C**, respectively). When we investigated cytokine production in each of these analyzed CD4^+^ T cell phenotypes, we observed that G-MDSCs reduce the frequency of IFN-γ and IL-17 in CD4^+^FOXP3^+^ T cells (**Figures 2D, E**), in contrast, IL-10 levels are similar across the different conditions (**Figures 2D, E**). On the other hand, in CD4^+^RORyt^+^ T cells, the percentage of IFN-γ, IL-17, and IL-10 are similar across the different conditions (**Figures 2F, G**). Regarding CD4^+^T-bet^+^ T cells, we observed that both G-MDSCs and M-MDSCs diminish the frequency of IFN-γ and IL-17 (**Figures 2H, I**). Finally, we assessed the viability of CD4^+^ T cells. We observed that the viability of these cells is similar across different conditions (**Figure 2J**).

**Figure 2:**
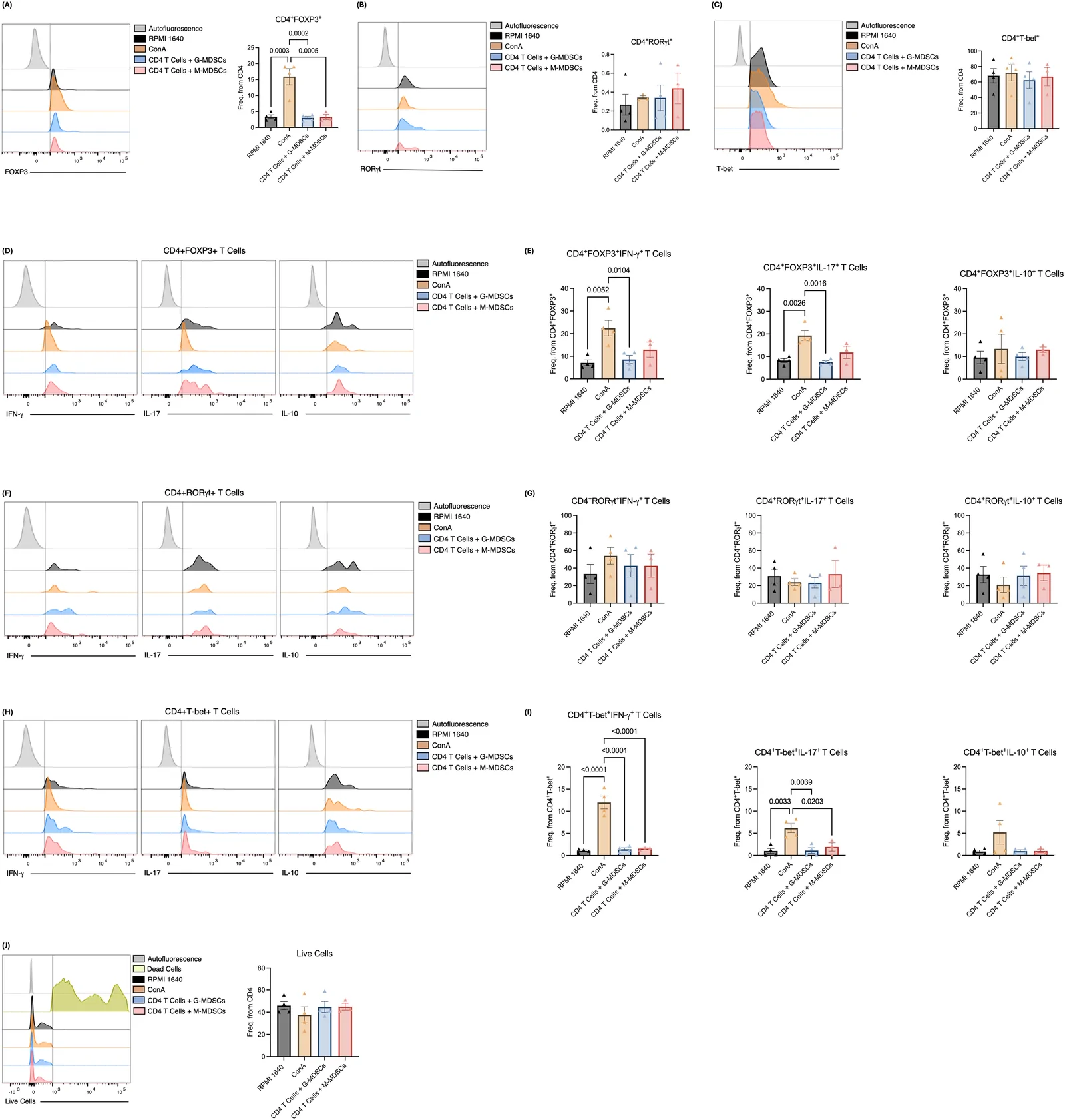
G-MDSC and M-MDSCs decrease the percentage of IFN- γ and IL-17 in CD4^+^ T cell phenotypes in SLE mice. Histogram of **(A)** FOXP3, **(B)** RORγt, and **(C)** T-bet, along with the percentage of transcription factors from CD4^+^ T cells. Histogram of IFN-γ, IL-17 e IL-10 from **(D)** FOXP3, **(F)** RORγt, and **(H)** T-bet in each of experimental conditions. Percentage of IFN-γ, IL-17, and IL-10 from **(E)** FOXP3, **(G)** RORγt, and **(I)** T-bet in each of experimental conditions. Histogram of **(J)** Live cells and the percentage of live cells from CD4^+^ T cells. Autofluorescence (Gray), CD4^+^ T cells in supplemented RPMI 1640 (black) n=4, CD4^+^ T cells stimulated with ConA (orange) n=4, CD4^+^ T cells + G-MDSCs (blue) n=4, and CD4^+^ T cells + M-MDSCs (red) n=3. Each histogram includes a gray vertical line that indicates the threshold for each of the markers. The statistical analysis used was one-way ANOVA post Tukey´s test, *p<* 0.05 are considered significant.

Once the selection and functionality of the MDSCs had been determined, we analyzed their presence in lung tissue. We observed the presence of both G-MDSCs and M-MDSCs in the lungs of SLE mice (**Figure 3A**). Similarly to the spleen, the frequency of both phenotypes was similar in both experimental groups (**Figures 3B, C**). In addition, the analyzed MDSCs also expressed Arg-1 and iNOS (**Supplementary Figures 4A–F**). Also, we analyzed F4/80 and CD11c expression in MDSCs present in the lungs (**Supplementary Figure 5**). Regarding G-MDSCs, we observed that the control group exhibited high heterogeneity for both markers, with F4/80^+^CD11c^+^ G-MDSCs as the most representative (**Supplementary Figure 5B**). In the SLE group, we observed that G-MDSCs CD11c^+^ were the most prevalent, followed by F4/80^-^CD11c^-^ G-MDSCs (**Supplementary Figure 5B**). Furthermore, when we evaluated M-MDSCs, we observed that in the control group virtually all of these cells were F4/80^+^ M-MDSCs (**Supplementary Figure 5C**), meanwhile in the SLE group, the most prevalent cells are M-MDSCs F4/80^+^ and F4/80^-^CD11c^-^ M-MDSCs (**Supplementary Figure 5C****).** On the other hand, when we examined in depth these phenotypes confirmed the expression of MHC-II in G-MDSCs and M-MDSCs from both experimental groups (**Figures 3D, G**). Regarding G-MDSCs, we observed that the percentage of MHC-II is approximately three times lower in SLE than in control mice (**Figure 3E**); nevertheless, the geometric mean of MHC-II is similar between both groups (**Figure 3F**). Meanwhile, M-MDSCs exhibit the opposite effect, since SLE mice show a higher percentage of MHC-II in the lung (**Figure 3H**) and an increase in the geometric mean of this molecule (**Figure 3I**). As with the general phenotype, we analyzed the presence of Arg-1 and iNOS in MDSCs expressing MHC-II and found that these markers are expressed in both experimental groups (**Supplementary Figures 4G–L**); however, MHC-II^+^ G-MDSC exhibit increased iNOS levels in SLE mice (**Supplementary Figure 4K**). These results suggest that the selected MDSCs might possess a immunosuppressive capacity over CD4^+^ T cells. In addition, the presence of APC-like MDSCs expressing MHC-II may display differential recruitment to SLE lungs.

**Figure 3:**
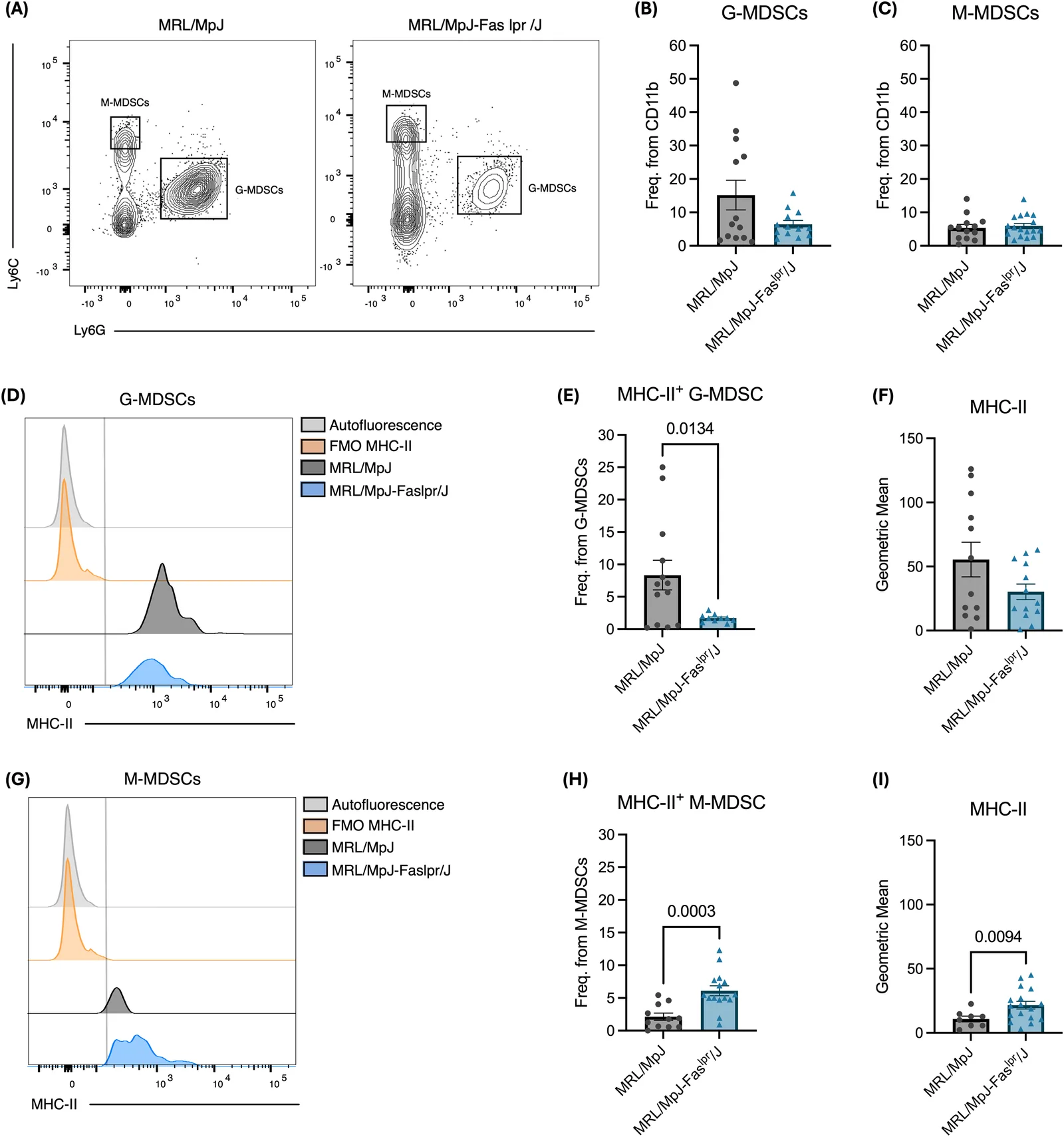
The presence of MDSCs in the lungs of SLE mice is characterized by an increase in the percentage and relative abundance of MHC-II on M-MDSCs. **(A)** Contour plot of G-MDSCs and M-MDSCs in the lungs of both experimental groups. Percentage of **(B)** G-MDSCs and **(C)** M-MDSCs from the CD11b population in each of the experimental groups. Histogram of MHC-II presence in **(D)** G-MDSCs and **(G)** M-MDSCs. The histograms show autofluorescence (gray), MHC-II FMO (orange), MRL/MpJ group (black), and MRL/MpJ-Fas^lpr^/J group (blue). Each histogram includes a gray vertical line that indicates the threshold for each of the markers. Percentage of MHC-II expression in **(E)** G-MDSCs and **(H)** M-MDSCs. Geometric mean of MHC-II in **(F)** G-MDSCs and **(I)** M-MDSCs. MRL/MpJ (black circles) n=8-14, MRL/MpJ-Fas^lpr^/J (blue triangles) n=10-17. The statistical analysis used was Welch’s t-test, *p<* 0.05 are considered significant.

### 3.3 MDSCs expressing MHC-II show decreased CD86 expression in the lungs of SLE mice

To investigate the potential APC-like phenotype MDSC expressing MHC-II in the lung, we evaluated the expression of costimulatory molecules, particularly CD80, CD86, and ICOSL, given their relevance in SLE development. We observed these three molecules in MDSCs expressing MHC-II in both experimental groups (**Figures 4A–H**). Specifically, in MHC-II^+^ G-MDSC cells, we observed similar CD80 percentage between the two experimental groups (**Figure 4A**) and similar geometric mean levels of this marker (**Supplementary Figure 6A**). On the other hand, the percentage of CD86-expressing and ICOSL-expressing MHC-II^+^ G-MDSCs were lower in SLE mice (**Figures 4B, C**). This reduction of CD86 and ICOSL expression was also reflected by the decreased geometric mean of these two costimulatory molecules (**Supplementary Figures 6B, C**). When we analyzed the frequency of CD80, CD86, and ICOSL on MHC-II^+^ G-MDSC in both experimental groups, we observed that in the lungs of SLE mice, CD80 is the most frequent costimulatory molecule in this MDSC phenotype (**Figure 4D**). Regarding MHC-II^+^ M-MDSC cells, the frequency of both CD80 and ICOSL is similar in both experimental groups (**Figures 4E, G**, respectively). Meanwhile, the frequency of CD86 is reduced in the SLE mice (**Figure 4F**). However, the geometric mean levels of these three costimulatory molecules in MHC-II^+^ M-MDSC cells are similar between the two experimental groups (**Supplementary Figures 6D–F**). On the other hand, when we analyze the pattern of co-stimulatory molecules on MHC-II^+^ M-MDSC, we observed the ratio among the three costimulatory molecules was similar (**Figure 4H**). These results suggest that MDSCs expressing MHC-II in the lungs of SLE mice also express a variety of co-stimulatory molecules which might contribute to APC function.

**Figure 4:**
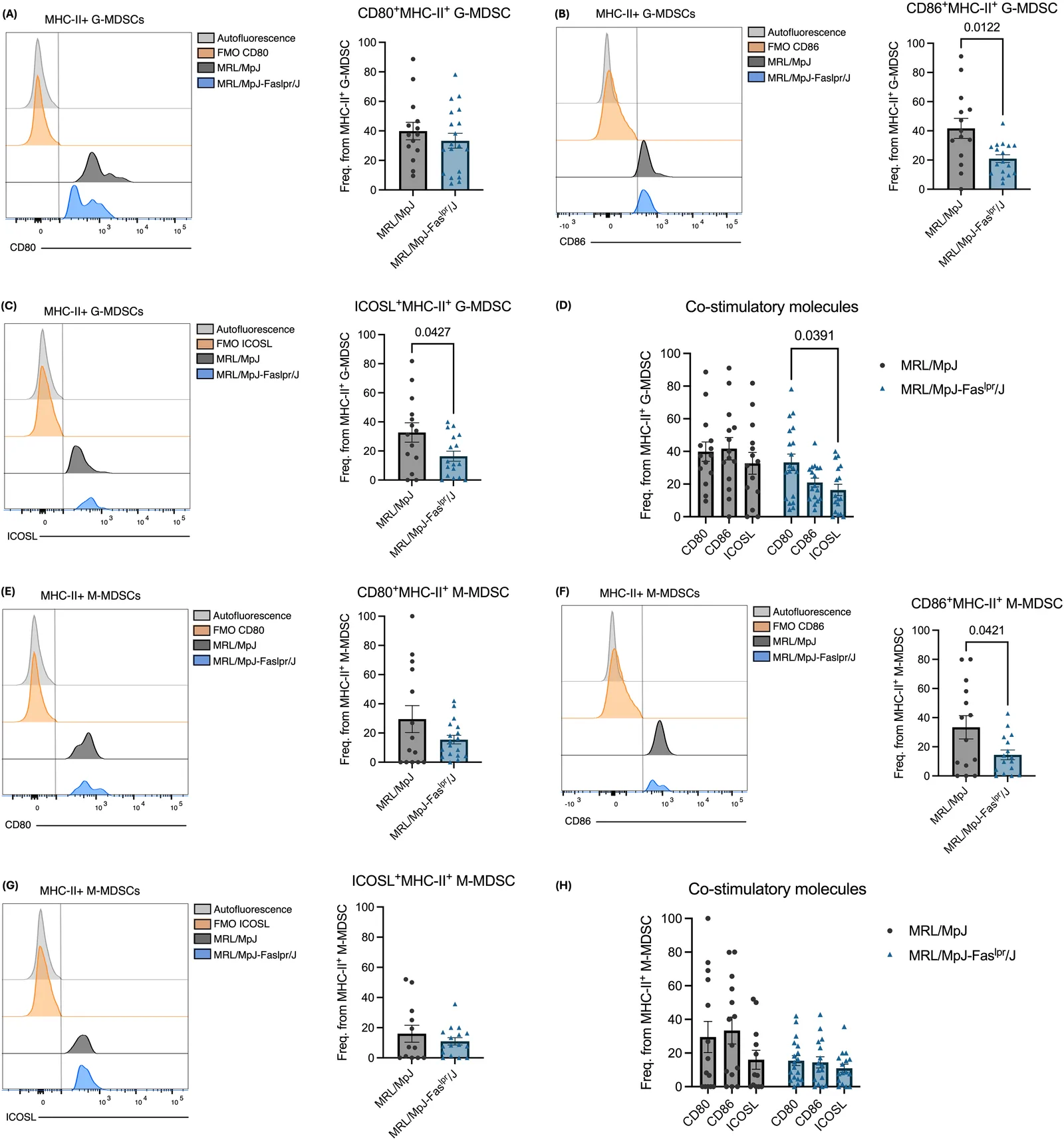
The percentage of CD86 present in both MDSC expressing MHC-II phenotypes is lower in SLE mice. The histogram and percentage of **(A)**, **(E)** CD80; **(B)**, **(F)** CD86; and **(C)**, **(G)** ICOSL in MHC-II^+^ G-MDSCs and MHC-II^+^ M-MDSCs, respectively. The histograms show autofluorescence (gray), co-stimulatory molecules FMO (orange), MRL/MpJ group (black), and MRL/MpJ-Fas^lpr^/J group (blue). Each histogram includes a gray vertical line that indicates the threshold for each of the markers. MRL/MpJ (black circles) n=12-14, MRL/MpJ-Fas^lpr^/J (blue triangles) n=16-19. The statistical analysis used was Welch’s t-test, *p<* 0.05 are considered significant. Analysis of the frequency of CD80, CD86, and ICOSL in **(D)** MHC-II^+^ G-MDSCs and **(H)** MHC-II^+^ M-MDSCs in both experimental groups. MRL/MpJ (black circles) n=12-14, MRL/MpJ-Fas^lpr^/J (blue triangles) n=16-19. two-way ANOVA post Tukey´s test, *p<* 0.05 are considered significant.

### 3.4 The lungs of SLE mice show a decreased frequency of CD4^+^FOXP3^+^ T cells

Since MDSCs are known to suppress the T-cell response we wanted to evaluate the status of T cells in the lungs of SLE mice. We observed that the lungs of SLE mice exhibit an increased frequency of CD3 cells (**Figure 5A**). Consequently, we analyzed the distribution of different T-cell phenotypes through a dimensional reduction analysis (UMAP) (**Supplementary Figure 7A**). The behavior of the clusters is directly related to the frequency of the T cells analyzed, since the frequency of CD4^+^ T cells is lower in the lungs of SLE mice (**Figure 5B**). Although CD8^+^ T-cell values are similar in both experimental groups, the SLE mice showed a downward trend in this population (**Supplementary Figure 7B**). Furthermore, the percentage of double-positive T cells (DP T cells) and double-negative T cells (DN T cells) are higher in the SLE mice (**Supplementary Figures 7C, D**). In particular, the distribution of T cells in the lungs of SLE mice is characterized by a widespread presence of DN T cells, which account for approximately 75% of all CD3^+^ cells, while the remaining 25% are distributed among the other T cell phenotypes (**Supplementary Figure 7E**). Further investigation of DN T cells revealed a higher abundance of B220^+^TCRαβ^+^ cells in the lungs of SLE mice (**Supplementary Figure 7F****).**

**Figure 5:**
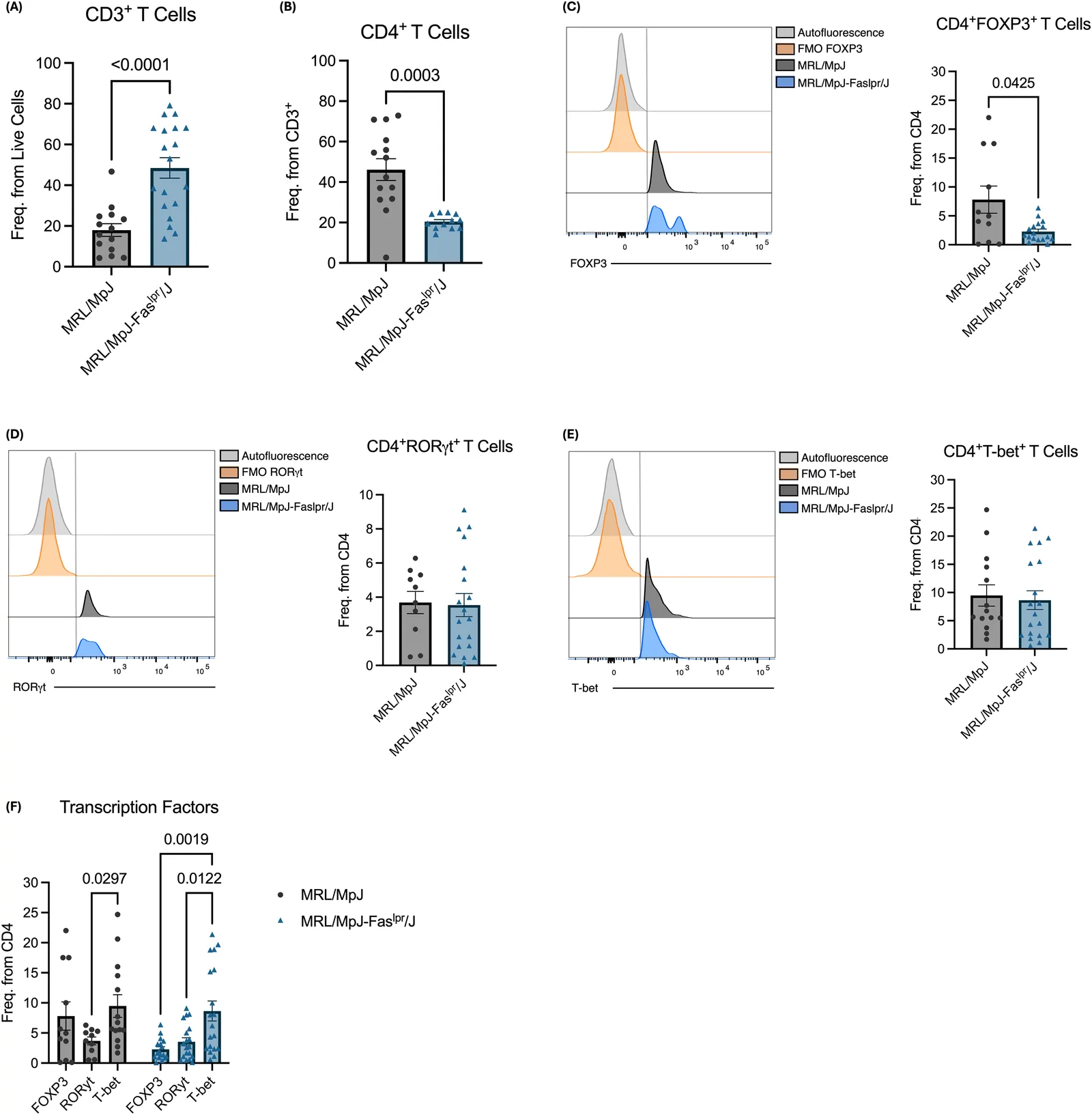
The phenotype of CD4^+^ T cells in the lungs of SLE mice is characterized by a reduction in anti-inflammatory profile. **(A)** Percentage of CD3^+^ T cells from live cells. **(B)** Percentage of CD4^+^ T Cells from CD3 cells. Histogram and percentage of CD4 T cells expressing **(C)** FOXP3, **(D)** RORγt, and **(E)** T-bet, respectively. The histograms show autofluorescence (gray), transcription factors FMO (orange), MRL/MpJ group (black), and MRL/MpJ-Fas^lpr^/J group (blue). Each histogram includes a gray vertical line that indicates the threshold for each of the markers. MRL/MpJ (black circles) n=10-14, MRL/MpJ-Fas^lpr^/J (blue triangles) n=17-19. The statistical analysis used was Welch’s t-test, *p<* 0.05 are considered significant. **(F)** Analysis of the frequency of FOXP3, RORγt, and T-bet in CD4^+^ T cells. MRL/MpJ (black circles) n=12-14, MRL/MpJ-Fas^lpr^/J (blue triangles) n=16-19. two-way ANOVA post Tukey´s test, *p<* 0.05 are considered significant.

Particularly, we are interested in evaluating CD4^+^ T cells due to their close relationship with antigen presentation mediated by MHC-II, which is expressed on MDSCs. To investigate CD4^+^ T cells in detail, we analyzed the master transcription factors linked to T_reg_, T_h17_ and T_h1_ cells. We observed a decrease in FOXP3 expression in CD4^+^ T cells from the lungs of SLE mice as compared to control mice (**Figure 5C**). On the other hand, both RORγt and T-bet showed similar percentage in CD4^+^ T cells in both experimental groups (**Figures 5D, E**). When we analyzed how the percentage of the three transcription factors relate to one another across the experimental groups, we observed that in the control group, T-bet is more frequent than RORγt, but is similar to the percentage of FOXP3 (**Figure 5F**). In contrast, in SLE mice, T-bet is the most common transcription factor in CD4^+^ T cells (**Figure 5F**). Altogether, these results show a reduction of FOXP3^+^ CD4^+^ Treg cells in the lungs of SLE mice, favoring a pro-inflammatory environment.

### 3.5 The lungs of SLE mice show a decrease in the frequency of IL-10 in CD4^+^RORγt ^+^ and CD4^+^T-bet^+^ T cells

To provide a more in-depth understanding of the different CD4^+^ T-cell phenotypes found in the lung of SLE mice, we analyzed IFN-γ, IL-17, and IL-10 by each of the CD4^+^ T helper cell subtypes. In both experimental groups and across the CD4^+^ T-cell phenotypes, we observed the presence of these three cytokines (**Figures 6A, F, K**). CD4^+^FOXP3^+^ T-cells exhibited similar percentage of IFN-γ, IL-17, and IL-10 expressing cells in both experimental groups (**Figures 6B–D**). However, when we analyzed how these cytokines are distributed among the phenotypes of CD4^+^ T cells, we observed that IFN-γ and IL-10 tend to be most prevalent cytokine in CD4^+^FOXP3^+^ T cells from the lungs of SLE mice (**Figure 6E**). On the other hand, CD4^+^ RORγt^+^ T cells show a similar frequency of IFN-γ and IL-17 between both experimental groups (**Figures 6G, H**), while the frequency of IL-10 in this cell population is decreased in SLE mice (**Figure 6I**). In contrast to what occurs with CD4^+^FOXP3^+^ T cells, when we analyzed the expression of these three cytokines in CD4^+^RORγt^+^ T cells, we observed that IL-17 and IL-10 was the most prominent in control mice; meanwhile in SLE mice, IL-17 IFN-γ tend to be most prevalent cytokine (**Figure 6J**). Finally, CD4^+^T-bet^+^ T cells exhibited a similar frequency of IFN-γ in both experimental groups (**Figure 6L**); however, the frequency of both IL-17 and IL-10 is decreased in SLE mice (**Figures 6M, N**). In this phenotype, IFN-γ and IL-17 were identified as tend to be most prevalent cytokine in SLE mice (**Figure 6O**). These results suggest that in the lungs of SLE mice, CD4^+^RORγt^+^ and CD4^+^T-bet^+^ T cells, due to their reduced production of anti-inflammatory IL-10 coupled with a predominance of pro-inflammatory cytokines IFN-γ and IL-17, may contribute to the inflammatory environment in lung tissue and disease progression.

**Figure 6:**
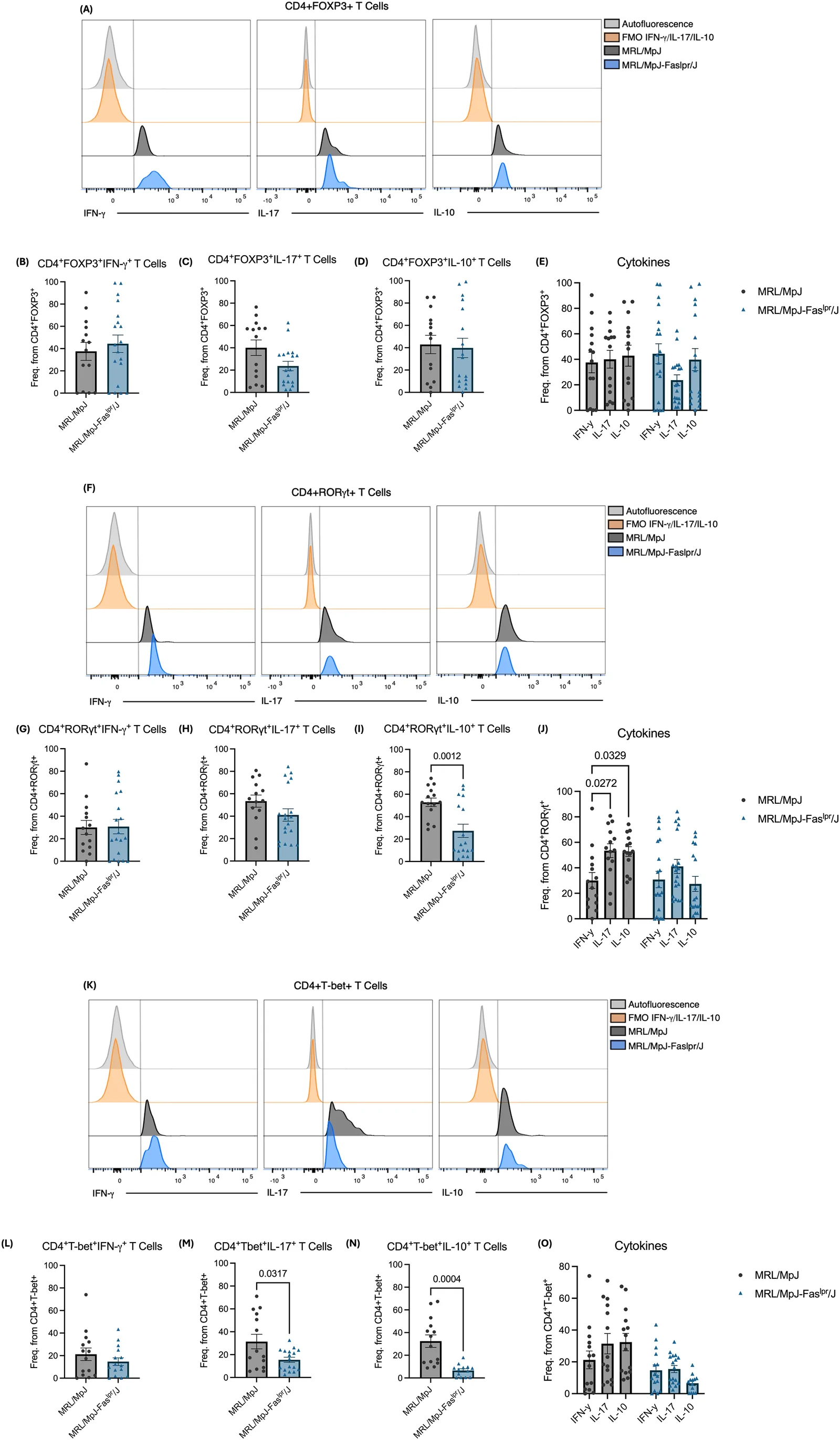
Both CD4^+^RORγt^+^ and CD4^+^T-bet^+^ T cells produce IL-10 at a lower percentage in the lungs of SLE mice. Histogram of **(A)** FOXP3, **(F)** RORγt, and **(K)** T-bet in CD4^+^ T Cells. The histograms show autofluorescence (gray), cytokines FMO (orange), MRL/MpJ group (black), and MRL/MpJ-Fas^lpr^/J group (blue). Each histogram includes a gray vertical line that indicates the threshold for each of the markers. Percentage of IFN-γ, IL-17, and IL-10 in CD4^+^ T cells expressing **(B–D)** FOXP3, **(G–I)** RORγt, and **(L–N)** T-bet. MRL/MpJ (black circles) n=14, MRL/MpJ-Fas^lpr^/J (blue triangles) n=15-19. The statistical analysis used was Welch’s t-test, *p<* 0.05 are considered significant. Analysis of the frequency of IFN-γ, IL-17, and IL-10 on CD4^+^ T cells expressing **(E)** FOXP3, **(J)** RORγt, and **(O)** T-bet in both experimental groups. MRL/MpJ (black circles) n=14, MRL/MpJ-Fas^lpr^/J (blue triangles) n=15-19. two-way ANOVA post Tukey´s test, *p<* 0.05 are considered significant.

### 3.6 Co-expression of MHC-II and costimulatory molecules on G-MDSCs positively correlates with pro-inflammatory CD4^+^ T cell response in lungs of SLE mice

Once we had analyzed the presence of MDSCs, MHC-II expression, and the distribution of CD4^+^ T cells in the lungs of SLE mice, we performed correlation analysis to assess potential associations between these cell populations. Regarding MDSCs, in the lungs of control mice, we observed a positive trend in the correlation between M-MDSCs and CD4^+^T-bet^+^IFN-γ^+^ T cells (**Figure 7A**). In contrast, in the lungs of SLE mice, we observed that M-MDSCs showed a more pronounced positive correlation with CD4^+^FOXP3^+^IL10^+^ and CD4^+^RORγt^+^IL-10^+^ T cells (**Figure 7B**). On the other hand, in lungs of control mice, we observed a positive correlation between MHC-II^+^ICOSL^+^ G-MDSCs and CD4^+^FOXP3^+^ T cells (**Figure 7A**). Likewise, regardless of MDSC MHC-II phenotype and the co-stimulatory molecules present on their surface, we found a very pronounced trend in which these phenotypes correlate positively with CD4^+^FOXP3^+^ T cells and CD4^+^T-bet^+^ T cells (**Figure 7A**). Meanwhile, in the lungs of SLE mice, MHC-II^+^ G-MDSC cells expressing CD80, CD86, or ICOSL show a positive correlation with CD4^+^FOXP3^+^IFN-γ^+^, CD4^+^FOXP3^+^IL-17^+^, CD4^+^RORyt^+^, CD4^+^RORyt^+^IFN-γ^+^, and CD4^+^RORyt^+^IL-17^+^ T cells (**Figure 7B**). Moreover, we analyzed how these MDSCs correlate with histopathological parameters in lungs of SLE and control mice. We observed that MHC-II^+^ G-MDSC cells expressing CD80, CD86, or ICOSL show a positive correlation with number of inflammatory foci in both experimental groups (**Supplementary Figures 8C, D**). Furthermore, in SLE mice, CD80^+^MHC-II^+^ M-MDSC correlates with inflammatory cellularity (**Supplementary Figure 8D**). Regarding cytokines, the presence of MHC-II^+^ G-MDSC and MHC-II^+^ICOSL^+^ G-MDSC correlates with the concentration of IL-17 in lung (**Supplementary Figure 8D**). These results suggest that MHC-II^+^ GMDSC cells expressing different costimulatory molecules could promote the differentiation of CD4^+^ T cells that produce proinflammatory cytokines, thereby contributing to the development and progression of lung inflammation associated with SLE.

**Figure 7:**
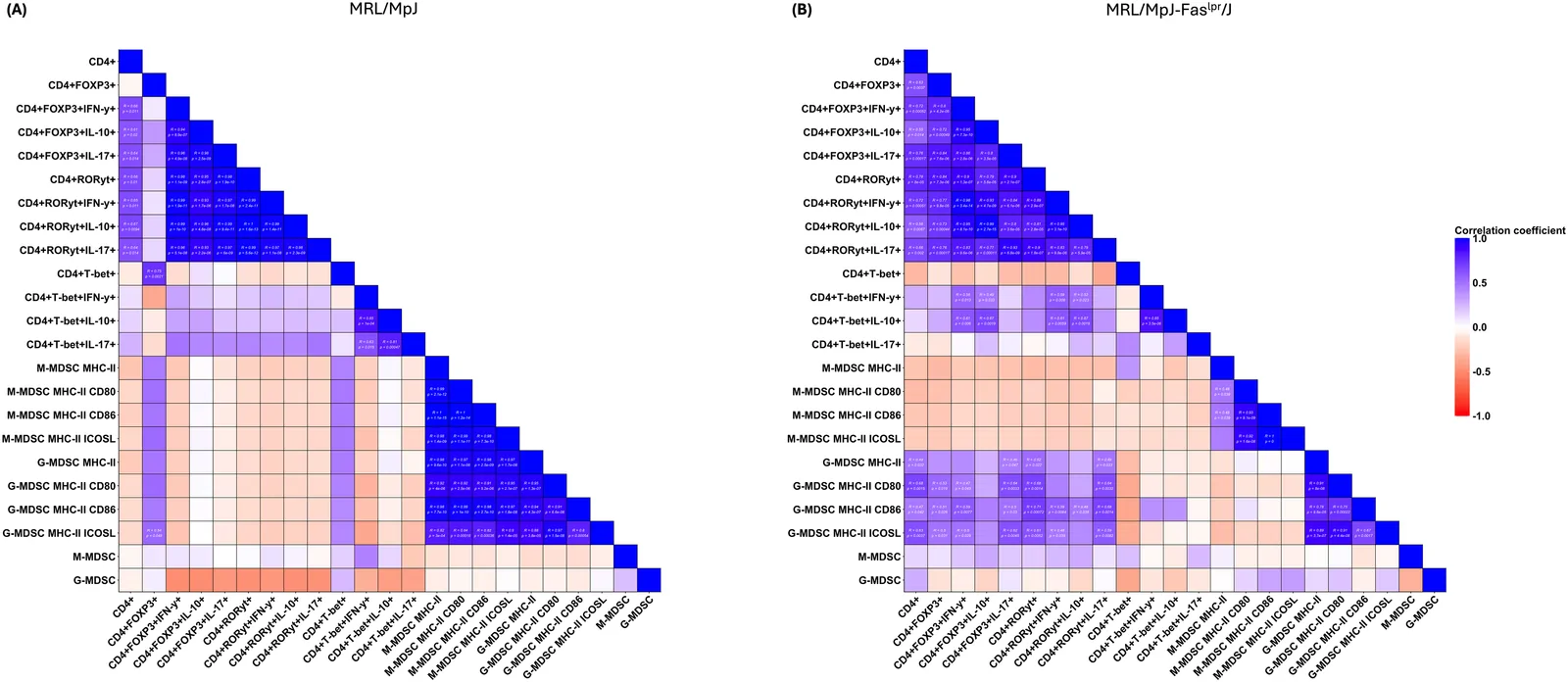
The phenotypes of MHC-II G-MDSCs correlate with T-cell variants that produce pro-inflammatory cytokines. The correlation analysis was performed based on the frequency of CD11b and CD3 cells, respectively, for each phenotype. Correlation between MDSCs, MHC-II^+^ MDSCs and CD4^+^ T cells in **(A)** MRL/MpJ and **(B)** MRL/MpJ-Fas^lpr^/J. The graphs show the correlation coefficient, which ranges from 1 (positive, blue) to -1 (negative, red). Moreover, significant correlations are indicated by the p-value and the correlation coefficient for each box. The number of observations is 14 for MRL/MpJ and 19 for the MRL/MpJ-Fas^lpr^/J. The statistical analysis used was Pearson’s correlation. *p<* 0.05 are considered significant.

## 4 Discussion

In our study, we used adult MRL/MpJ-Fas^lpr^/J mice from 16 weeks of age onward as a model of systemic lupus erythematosus (SLE), since the disease is already fully established in this model at that stage (26). Moreover, animals in this age group are in the adult stage (27, 28), which make immunological and histological analysis of the lung optimal, as clinical manifestations involving the lungs are most commonly seen in adult SLE patients (7). The SLE mice utilized in our study exhibited clinical manifestations characteristic of the disease (**Supplementary Figure 2**), which are observed in both patients and murine models of SLE, including splenomegaly (29, 30), proteinuria (26, 31), and elevated levels of anti-dsDNA antibodies (32–34). For this reason, we have concluded that our animals display a phenotype consistent with systemic lupus erythematosus (SLE).

Our findings reveal changes in the pulmonary immune landscape in SLE. First, we demonstrate that the pulmonary environment in SLE mice is dysregulated, characterized by a predominance of pro-inflammatory cytokines (**Figure 1**). This loss of homeostasis in the lung could promote the development of pulmonary manifestations, given that IL-17 and TNF contribute to both the development of chronic lung diseases and to inflammation of bronchi and alveoli (35, 36). In addition, IL-6 has been established as a marker of organ-specific damage in SLE (37, 38). This is further supported by the decrease in IL-10 (**Figure 1B**), a protective cytokine against pulmonary manifestations in SLE (39), which could indicate that tissue damage is being induced. These findings are consistent with the increased histopathological score in the lungs of SLE mice (**Figures 1E–K**). The primary factor contributing to this parameter is cellular infiltration. Although the specific infiltrating cell populations were not identified in the present histological analysis, these findings are histologically compatible with inflammatory leukocytic infiltration and are consistent with previously described pulmonary inflammatory involvement in SLE (40, 41). The pro-inflammatory cytokines evaluated (IL-17, TNF, IL-6) may also be involved in this process, since TNF and IL-6 can induce pulmonary permeability, leading to cellular infiltration (42–44). Furthermore, IL-17 in SLE promotes the migration of monocytes, neutrophils, and T cells to inflamed tissue (45). Additionally, IL-17 induces IL-6 production in SLE (45), which could be causing a continuous cycle of infiltration and inflammation in the tissue, promoting the development of pulmonary manifestations related to SLE.

MDSCs isolated from the spleens of SLE mice exhibit the immunosuppressive function characteristic of these cells, since they reduce the production of IFN-γ and IL-17 in both CD4^+^FOXP3^+^ and CD4^+^T-bet^+^ T cells (**Figure 2**). This same effect has been reported in the literature, in which both G-MDSCs and M-MDSCs can decrease the production of IFN-γ and IL-17 in T cells (46–49). Although we observe that the viability percentage of CD4^+^ T cells is similar across the different conditions (**Figure 2J**), this does not exclude the possibility that MDSCs possess an immunosuppressive function. Since the loss of viability of CD4^+^ T cells is not a mechanism of immunosuppression mediated by MDSCs, these cells prefer other pathways to modulate the immune response (50, 51). Given this, the selection of MDSCs from the spleen was the same as that applied to the lungs of SLE mice, we can infer that both G-MDSCs and M-MDSCs found in lung tissue also could exhibit this immunosuppressive activity. Moreover, to support this idea, it has been reported that in response to a chronic condition, MDSCs can migrate from the spleen to lung tissue (52, 53). On the other hand, it should be noted that F4/80 and CD11c expression in MDSCs (**Supplementary Figure 5**) is not a disqualifying factor, even though they are classic markers of other innate cells. This is because it has been reported that MDSCs can express F4/80 (54, 55) Even in cancer, it has been observed that M-MDSCs expressing this marker often subsequently differentiate into tumor-associated macrophages (TAMs) (56). However, SLE does not express TAMs; therefore, the expression of this marker could indicate the plasticity of these cells to shift from a G-MDSC to an M-MDSC, as has been observed in *Staphylococcus aureus* infection (57). Furthermore, CD11c expression in MDSCs has been associated with an increase in the immunosuppressive function of these cells (58).

In the SLE context, MDSCs have gained greater relevance in recent years, even being the focus of clinical studies (59). However, the presence of MDSCs in the lungs has not been thoroughly investigated; only that these cells are involved in the pathogenesis of diffuse pulmonary hemorrhage in SLE mice (60) has been reported. Therefore, the presence of MDSCs expressing MHC-II in the lungs of SLE mice (**Figures 3D–I**) suggest that the inflammatory environment in the lung tissue may be modulating MHC-II expression in these cells. This is supported by research on EAE, in which MDSCs exposed to an IFN-γ-induced inflammatory environment showed an increase in MHC-II expression (16), this increase was further enhanced in the M-MDSC phenotype, as shown in our results (**Figures 3G–I**).

When we decided to investigate the costimulatory molecules in MDSC MHC-II, we found that both phenotypes express CD80, CD86, and ICOSL (**Figures 4A–H**). It is known that in SLE, the B7 complex molecules (CD80/CD86) and ICOSL promote the development of the disease through antigen presentation and activation of humoral immunity, thereby increasing IgG antibody production (18, 61). However, both in MHC-II^+^ G-MDSCs and MHC-II^+^ M-MDSCs, we observed a decrease in the frequency of CD86 (**Figures 4B, F**, respectively), which would be counterintuitive given that MDSCs MHC-II should exhibit an increase in this marker in the context of SLE. Nevertheless, it has been reported that an anti-inflammatory environment driven by TGF-β1 induces an increase in CD86 expression (62). Furthermore, in a systemic inflammatory context, CD86 has been shown to regulate IL-10 production, thereby triggering an anti-inflammatory environment (63). Even in cancer, it has been observed that CD86 can act as a double-edged sword, as it can induce the activation of T_reg_ (64). This may suggest that, under non-pathological conditions, increased CD86 expression in MHC-II MDSCs may be inducing an immunoregulatory environment. We also observed a decrease of ICOSL in MHC-II^+^ G-MDSCs from the lungs of SLE mice (**Figure 4C**); this may be because this molecule could also be exerting a dual effect. In autoimmune diseases such as SLE and rheumatoid arthritis (RA), ICOSL promotes an inflammatory response and disease progression (18, 65). Even in cancer, the interaction between ICOSL and the osteoponin ligand promotes angiogenesis and tumor metastasis (66, 67). On the other hand, it has also been observed that the ICOS/ICOSL pathway can counteract systemic inflammation (68). It has been reported that in kidney disease, ICOSL interacts with the αvβ3 integrin in podocytes, which maintains glomerular filtration, thereby reducing proteinuria (69). Moreover, ICOSL expressions in macrophages can induce an M2 response, producing an anti-inflammatory environment that repairs tissue damage (70). This ICOSL activity associated with a protective response could be present in the control mice. Nevertheless, SLE is characterized by chronic inflammation; it has been reported that an inflammatory environment characterized by high levels of IL-12 or IFN-γ can modulate MDSCs by enhancing their APC phenotype, thereby increasing the expression of MHC-II and CD80 (16, 71). In addition, MDSCs have been reported to acquire phagocytic capacity in response to tuberculosis (11), a capacity that plays a key role in their development into APCs. The presence of MDSCs expressing CD80, CD86, and MHC-II has been observed in autoimmune diseases such as EAE and RA; however, it has not been established whether they play a key role in the pathogenesis of these diseases (16, 72). Furthermore, it is known that in these diseases, the presence of an inflammatory environment promotes the expression of these markers in MDSCs (16, 72, 73).

The decrease in the frequency of CD4^+^ T (**Figure 5B**) cells in the lungs of SLE mice is due to the appearance of DN and DP T cells in this tissue (**Supplementary Figure 7**). This effect has already been reported in SLE (74–76). However, when we examined the phenotypes of CD4^+^ T cells, we observed that FOXP3 expression was reduced in SLE mice (**Figure 5C**). It is known that, in SLE, a decrease in FOXP3 T cells is positively correlated with disease activity (77). This decrease in FOXP3^+^CD4^+^ T cells could also be explained by the chronic inflammatory condition that can change the phenotype of T cells, leading to the loss of expression of this transcription factor and the secretion of pro-inflammatory cytokines (78). On the other hand, although the expression levels of RORγt and T-bet are similar between the experimental groups (**Figures 5D, E**), the relative distribution of T-bet are more abundant in the lungs of SLE mice (**Figure 5F**), which could be associated with pro-inflammatory profiles (79, 80). When we analyze cytokine production, these cells show decrease IL-10 levels in SLE mice (**Figures 6I, N**). It is known that, in response to chronic stimulation, phenotypes such as T_h1_ and T_h17_ can begin to produce this cytokine (81, 82). Even to prevent the development of diseases, a dynamic relationship exists between T_h17_ cells and T_reg_ cells (83). Therefore, in a non-pathological condition (control group), all these mechanisms are functioning properly to maintain immune homeostasis in the lung tissue. We can also observe that CD4^+^ T cells expressing T-bet exhibit decreased in IL-17 expression in SLE animals (**Figure 6M**). This may be due to the cytokine environment present in this tissue, as the presence of pro-inflammatory cytokines can modulate the phenotype of CD4^+^ T cells (84, 85). In this case, the change in the frequency of IL-10 and IL-17 could be due to phenotypic polarization toward a T_reg_ phenotype; on the other hand, T_h17_ cells could shift toward a T_h1_ phenotype, which could indicate that the inflammatory environment is altering CD4^+^ T cells to induce a stronger inflammatory response. However, when evaluating these CD4^+^ T cell phenotypes, we cannot overlook the *Fas* mutation present in these SLE mice, as the Fas pathway is known to play an important role in modulating the response in these cells (86, 87).

When we analyzed the correlations between MDSCs and CD4^+^ T cells, we observed that in the lungs of the control group there is a positive correlation between M-MDSCs and CD4^+^T-bet^+^IFN-γ^+^ T cells (**Figure 7A**); furthermore, when we evaluated the correlations exhibited by MDSCs expressing MHC-II, we observed that both MHC-II^+^ G-MDSC and MHC-II^+^ M-MDSC expressing various costimulatory molecules correlated with CD4^+^FOXP3^+^ T cells and CD4^+^T-bet^+^ T cells (**Figure 7A**). Therefore, under these conditions, MDSCs MHC-II may be exerting an immunoregulatory effect due to their association with CD4^+^FOXP3^+^ T cells (78). Also, these cells correlate with CD4^+^T-bet^+^ T cells, which can likewise exert an anti-inflammatory effect, since in pulmonary diseases these cells reduce T_h17_ production (88, 89). This effect is evident in a slight negative trend in our results (**Figure 7A**). On the other hand, in SLE mice, we observed that M-MDSCs tend to correlate positively with CD4^+^FOXP3^+^IL10^+^ and CD4^+^RORyt^+^IL10^+^ T cells (**Figure 7B**). This would be associated with an anti-inflammatory response, a phenomenon consistent with what has been described in the literature, since MDSCs induce Tregs (90, 91). However, when we evaluated specific MDSC phenotypes, specifically those expressing MHC-II, we observed that G-MDSCs that express MHC-II and various costimulatory molecules show a positive correlation with both anti- and pro-inflammatory phenotypes of CD4^+^ T cells (**Figure 7B**); nevertheless, the most significant correlations are with respect to CD4^+^ T cells that have a potential pro-inflammatory phenotype, as they produce IL-17 or IFN-γ (**Figure 7B**). In contrast to what occurs in the control group, MDSCs MHC-II could be promoting a pro-inflammatory environment, since IL-17 and IFN-γ are cytokines that promote SLE pathogenesis (92–94). Moreover, it has been reported in the literature that MDSCs can promote the proliferation and response of T_h17_ cells (24, 95). It should also be noted that G-MDSCs expressing MHC-II and various costimulatory molecules represent a small population, as the percentage of MHC-II-expressing G-MDSCs is lower in the lungs of SLE mice (**Figure 3E**). However, this does not mean that their potential immune function is reduced, since small immune populations such as ILCs, MAITs, and NK cells exert fundamental and substantial immunological effects in the organism (96–99). The correlation we observed in the lungs of SLE mice between MHC-II^+^ G-MDSCs and CD4^+^ T cells that produce pro-inflammatory cytokines (**Figure 7B**) is consistent with a phenomenon described in inflammatory bowel diseases (IBD) (100). In these conditions, cells like G-MDSCs have been observed to exhibit an APC phenotype, which promotes T-cell activation and the secretion of pro-inflammatory cytokines (IL-1β, IFN-γ, TNF-α, IL-17, among others), thereby driving the progression of the disease (100). Therefore, it would be interesting to evaluate in the future the role played by MDSCs with an APC phenotype in autoimmune diseases and to determine whether their effect is tissue-specific or systemic.

The limitations of our study are based on immunosuppression assays not being performed with pulmonary MDSCs, but rather with MDSCs from the spleen. Because the current technique results in a low yield of MDSCs (**Supplementary Figure 9**), we speculate that the yield in the lung would be even lower, given that it is the target organ where these cells migrate. To perform this protocol in the lung at present, we would need a high number of animals, which would conflict with the 3Rs. Additionally, we were unable to confirm the direct effect of different MHC-II-expressing MDSC phenotypes on CD4^+^ T cells in lung tissue. We also did not determine how the presence of different costimulatory molecules might affect this interaction. It remains to be evaluated in future studies whether these cells can act as APCs beyond merely presenting a similar phenotype. Furthermore, we were unable to directly assess how environmental cytokines might modulate the expression of markers associated with the APC phenotype in MDSCs. However, our results have a significant impact on the field of interest, since, in summary, our results indicate that the lungs of SLE mice present an exacerbated pro-inflammatory environment along with increased cellular infiltration, which may be creating a vicious cycle of inflammation and progressive infiltration. This alteration in the pulmonary environment could be modulating the behavior of MDSCs on this tissue, mainly by inducing the expression of MHC-II, CD80, CD86, and ICOSL, thereby generating an APC-like phenotype. In the lungs of SLE mice, the presence of MHC-II^+^ G-MDSC cells expressing various costimulatory molecules correlates with CD4^+^ T cells that exhibit a pro-inflammatory phenotype due to their production of IL-17 and IFN-γ. Therefore, the APC-like phenotype that MDSCs acquire could be giving rise to a potential new function for these cells, which are characterized by an immunosuppressive response This could induce activation, polarization, and/or an inflammatory response in CD4^+^ T cells. This could promote the development of an inflammatory environment and tissue damage, creating a potential vicious cycle that leads to disease progression.

## Data Availability

The raw data supporting the conclusions of this article will be made available by the authors, without undue reservation.
