# Supplementary material for: MHC-II+ G-MDSC expressing CD80, CD86, or ICOSL are associated with pro-inflammatory CD4+ T cells phenotypes in the lungs of lupus-prone mice
Source: Front Immunol. 2026 Oct 26;17:1891967. doi: 10.3389/fimmu.2026.1891967 (PMC13745663; doi:10.3389/fimmu.2026.1891967)
Supplement: Supplementary file 1 [file Supplementaryfile1.docx]

Supplementary Materia

**Supplementary Figure 1. Gating Strategy. (A)** The gating strategy for CD4+ T cells, the populations of interest are highlighted in red. FMO from markers for **(B)** T Cells, and **(C)** MDSCs.

**Supplementary Figure 2. Increased pathological parameters in MRL/MpJ-Fas^lpr^/J mice.** **(A)** Photograph of the spleen from both experimental groups, including length (cm). MRL/MpJ (black circles) n=10, MRL/MpJ-Fas^lpr^/J (blue triangles) n=14. The statistical analysis used was Welch’s t-test, *p<* 0.05 are considered significant. **(B)** Analysis of proteinuria based on the score described by Uribe, FR., *et al* (14). MRL/MpJ (black circles) n=32, MRL/MpJ-Fas^lpr^/J (blue triangles) n=29. The statistical analysis used was the Mann-Whitney test, p< 0.05 are considered significant. **(C)** Concentration of anti-dsDNA antibodies in the serum of both experimental groups. MRL/MpJ (black circles) n=21, MRL/MpJ-Fas^lpr^/J (blue triangles) n=17. The statistical analysis used was Welch’s t-test, *p<* 0.05 are considered significant.

**Supplementary Figure 3. M-MDSCs from the spleens of SLE mice show an increased percentage of iNOS. (A)** Contour plot of G-MDSCs and M-MDSCs in spleen of both experimental groups**. (B)** Percentage of G-MDSCs and M-MDSCs from the CD11b population in each of the experimental groups. Histogram of **(C)** Arg-1 and **(E)** iNOS presence in MDSCs. The histograms show autofluorescence (gray), Arg-1/iNOS FMO (orange), MRL/MpJ group (black), and MRL/MpJ-Fas^lpr^/J group (blue). Each histogram includes a gray vertical line that indicates the threshold for each of the markers. Percentage of **(D)** Arg-1 and **(F)** iNOS in MDSCs. MRL/MpJ (black circles) n=7, MRL/MpJ-Fas^lpr^/J (blue triangles) n=13. The statistical analysis used was Welch’s t-test, *p<* 0.05 are considered significant.

**Supplementary Figure 4. G-MDSCs expressing MHC-II show an increased percentage of iNOS in the lungs of SLE mice.** Histogram of **(A, G)** Arg-1 and **(D, J)** iNOS in MDSCSs and MDSCs expressing MHC-II. The histograms show autofluorescence (gray), Arg-1/iNOS FMO (orange), MRL/MpJ group (black), and MRL/MpJ-Fas^lpr^/J group (blue). Each histogram includes a gray vertical line that indicates the threshold for each of the markers. Percentage of **(B, C, H, I)** Arg-1 and **(E, F, K, L)** iNOS in MDSCs and MDSCs expressing MHC-II. MRL/MpJ (black circles) n=14, MRL/MpJ-Fas^lpr^/J (blue triangles) n=15–19. The statistical analysis used was Welch’s t-test, *p<* 0.05 are considered significant.

**Supplementary Figure 6. The selected MDSCs express markers such as F4/80 and CD11c. (A)** Gating strategy based on a data concatenation that represents the process from total cells to the selection of the cells of interest. Target populations are highlighted in yellow. Analysis of the frequency of F4/80 (black), CD11c (blue), F4/80^-^CD11c^-^ (yellow), and F4/80^+^CD11c^+^ (emerald) in **(B)** G-MDSCs and **(C)** M-MDSCs. MRL/MpJ n=3, MRL/MpJ-Fas^lpr^/J n=3. The statistical analysis used was one-way ANOVA post Tukey´s test, *p<* 0.05 are considered significant.

**Supplementary Figure 6. The relative abundance of CD86 and ICOSL decreased in MHC-II^+^ G-MDSC in the lungs of SLE mice.** Geometric mean plots of **(A)** CD80, **(B)** CD86, and **(C)** ICOSL in MHC-II^+^ G-MDSC cells. Geometric mean plots of **(D)** CD80, **(E)** CD86, **(F)** ICOSL in MHC-II^+^ M-MDSCs. MRL/MpJ (black circles) n=8–13, MRL/MpJ-Fas^lpr^/J (blue triangles) n=12–18. The statistical analysis used was Welch’s t-test, *p<* 0.05 are considered significant.

**Supplementary Figure 7. Alterations in T-cell frequency in the lungs of SLE mice.** **(A)** Uniform Manifold Approximation and Projection (UMAP) based on a joint concatenation of both experimental groups. CD4^+^ T cells (Black), CD8^+^ T cells (Yellow), double-positive T cells (Red), and double-negative T cells (Blue). Frequency of T cells: **(B)** CD8, **(C)** double-positive (DP), and **(D)** double-negative (DN) cells in both experimental groups. MRL/MpJ (black circles) n=10–14, MRL/MpJ-Fas^lpr^/J (blue triangles) n=12–18. The statistical analysis used was Welch’s t-test, *p<* 0.05 are considered significant. **(E)** Analysis of the frequency of CD4 T Cells (black), CD8 T Cells (blue), DN T Cells (yellow), and DP T Cells (emerald) from CD3 cells. MRL/MpJ n=10–14, MRL/MpJ-Fas^lpr^/J n=12–18. The statistical analysis used was one-way ANOVA post Tukey´s test, *p<* 0.05 are considered significant. **(F)** Contour plot and percentage of B220+TCRb+ cells from DN T cells in both experimental groups. MRL/MpJ (black circles) n=3, MRL/MpJ-Fas^lpr^/J (blue triangles) n=3. The statistical analysis used was Welch’s t-test, *p<* 0.05 are considered significant.

**Supplementary Figure 8. MHC-II^+^ G-MDSC cells correlate with cell infiltration spots in the lungs of SLE mice.** The correlation analysis was performed based on the percentage MHC-II^+^ MDSCs and parameters evaluated in Section 3.1 in **(A)** MRL/MpJ and **(B)** MRL/MpJ-Fas^lpr^/J. The graphs show the correlation coefficient, which ranges from 1 (positive, blue) to -1 (negative, red). Moreover, significant correlations are indicated by the p-value and the correlation coefficient for each box. The number of observations is 7 for MRL/MpJ and 8 for the MRL/MpJ-Fas^lpr^/J. The statistical analysis used was Pearson’s correlation. *p<* 0.05 are considered significant.

**Supplementary Figure 9. Yield of G-MDSCs and M-MDSCs isolated from total splenocytes.** A total of 100,000,000 total splenocytes (Blue) were used to isolate G-MDSCs (Brown) and M-MDSC (grey).

**Supplementary Table 1. Histopathological score**

| **Parameter** | **Score** | **Definition** |
| --- | --- | --- |
| **Number of inflammatory foci** | **0** | No inflammatory spot observed |
|  | **1** | Focal involvement: 1–3, well-defined inflammatory foci |
|  | **2** | Mild multifocal involvement: 4–7 discrete inflammatory foci with preserved intervening parenchyma. |
|  | **3** | Moderate multifocal involvement: 8–11 inflammatory foci with partial loss of preserved intervening parenchyma. |
|  | **4** | Severe multifocal/extensive involvement: ≥12 inflammatory foci with marked loss of preserved intervening parenchyma |
| **Mean inflammatory focus area (µm²)** | **0** | No appreciable expansion of inflammatory foci (≤29,753µm²) |
|  | **1** | Minimal expansion of inflammatory foci (29,754–39,022µm²) |
|  | **2** | Mild expansion of inflammatory foci (39,023–48,291µm²) |
|  | **3** | Moderate expansion of inflammatory foci (48,292–57,560µm²) |
|  | **4** | Severe expansion of the inflammatory foci (>57,560µm²) |
| **Inflammatory cellularity (cells/focus)** | **0** | No appreciable increase in inflammatory cellularity (≤432.3 cells/focus) |
|  | **1** | Minimal increase in inflammatory cellularity (432.4–580.9 cells/focus) |
|  | **2** | Mild increase in inflammatory cellularity (581.0–729.5 cells/focus) |
|  | **3** | Moderate increase in inflammatory cellularity (729.6–878.1 cells/focus) |
|  | **4** | Severe increase in inflammatory cellularity (>878.1 cells/focus) |
| **Alveolar collapse** | **0** | No evidence of alveolar collapse (77.8% alveolar airspace) |
|  | **1** | Minimal alveolar collapse (68.6–77.7% alveolar airspace) |
|  | **2** | Mild alveolar collapse (59.4–68.5% alveolar airspace) |
|  | **3** | Moderate alveolar collapse (50.2–59.3% alveolar airspace) |
|  | **4** | Severe alveolar collapse (<50.2% alveolar airspace) |
